# Giant prostatic calculi

**DOI:** 10.11604/pamj.2013.14.69.2376

**Published:** 2013-02-19

**Authors:** Mohammed Najoui, Abdelmounaim Qarro, Abdelghani Ammani, Mohammed Alami

**Affiliations:** 1 Departement of urology of Military hospital, Meknès, Maroc

**Keywords:** Prostatic calculi, endogenous, exogenous

## Abstract

Prostatic parenchymal calculi are common, usually incidental, findings on morphological examinations. They are typically asymptomatic and may be present in association with normal glands, benign prostatic hyperplasia, and prostate cancer. However giant prostatic calculi are rare. Less than 20 cases have been reported in the literature. We present the case of a 35-year-old man with two giant prostatic calculi that replaced the entire gland. He underwent an open cystolithotomy, two giant stones were removed from the prostate, and we used a lithotripsy in situ for extraction of stone fragments.

## Introduction

Prostatic calculi correspond to the calculations developed in the prostate tissue itself and are distinguished from calculations locked in the prostatic urethra, bladder or renal origin. They are classified into two groups according to their origin, endogenous or primitives are formed from prostatic secretions and exogenous or secondary are formed within the prostatic ducts from constituents of the urine. They are rare in children but common in men over 50 years, with incidence increasing with age; they are frequently associated with BPH or chronic prostatitis.

We report a case of patient admitted for dysuria that clinical and radiological examination is revealed the presence of two calculations employing both prostatic lobes in full.

## Patient and observation

**Figure 1:**
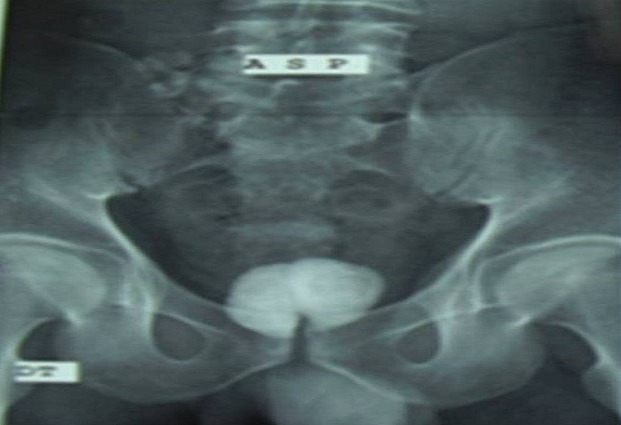
KUB showing two giant prostatic calculi

**Figure 2:**
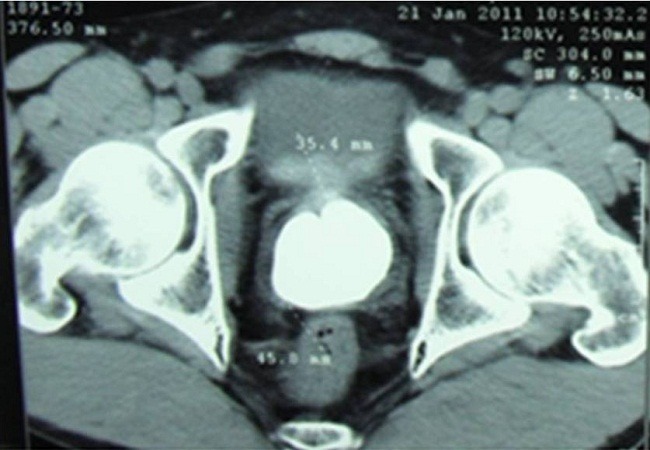
Nonenhanced CT Scan showing replacement of prostate gland with calculi

**Figure 3:**
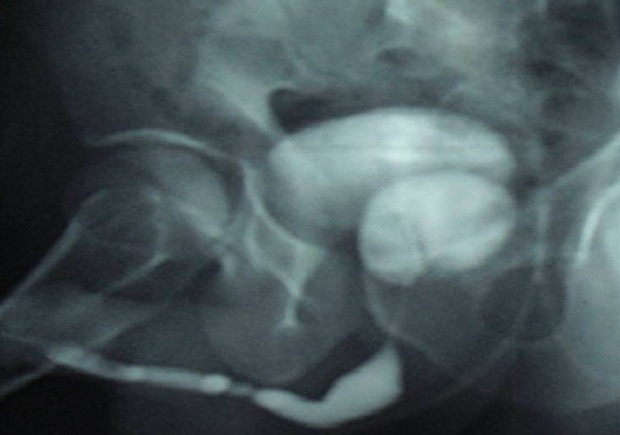
Urethral stenosis

**Figure 4:**
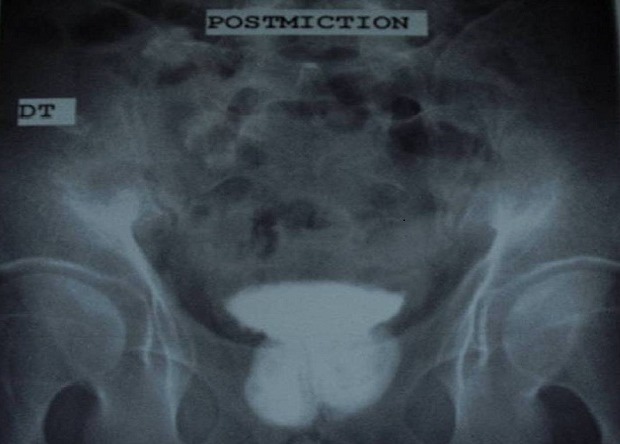
Significant post voiding urine

This is a 35-year-old patient, unmarried, which has since childhood voiding obstruction with recurrent urinary infection without other associated signs especially ejaculatory difficulties or hemospermia. Five months ago the patient had several episodes of hematuria prompting its consultation. The examination for admission did not find a distended bladder or palpable masses or lumbar contact. On digital rectal examination there is a stony suspect prostate being. The rest of the examination is unremarkable. An assessment was requested. An examination of the kidneys, ureter, and bladder (KUB) (Figure 1) and CT scan (Figure 2) were performed and revealed two prostatic stones replaced the entire gland. The retrograde cystography objectified suspicious urethral stenosis (Figure 3), however, a significant post void urine volume was highlighted (Figure 4). Blood and urine calcium phosphate levels were normal. An urethrocystoscopy showing normal urethra and no evident urethral stenosis, two giant prostatic calculi and bladder with marked trabeculation, biopsy of prostate and bladder revealed no abnormalities. The patient had a cystolithotomy; after a lower midline incision, opening of the bladder wall between two landmark son was put in place the spacers in the Hryntschak, then we have made pericervical incision we tried to extract the 2 calculations employing both prostate lobes, however their extraction through the pericervical incision was impossible, so we used a lithotripsy in situ. Infrared stone analysis revealed that it was composed of calcium phosphates.

## Discussion

Less than 20 cases of giant prostatic calculi have been reported in the literature. They occur more frequently in younger men, unlike microscopic prostatic calculi, which are usually seen in men older than 50 years [1]. They can be classified into “endogenous” or primitives, “exogenous” or secondary. Endogenous calculations can be formed from direct precipitation of elements present in prostatic secretions stasis that results from obstruction, inflammation and chronic infection of the prostate ducts. When the stones exogenous, they are formed from constituents of the urine, as shown by crystallographic studies of prostatic calculi [2]. In fact hypercalciuria is more often found in patients with large prostatic calculi [3]. Prostatic calculi have been reported in patients with alkaptonuria [4]. In this disease, a rare cause of prostatic calculi, excess homogentisic acid in urine polymerizes on contact with alkaline prostatic secretions, thus forming the nucleus for the calcium precipitation.

Prostatic calculi can be single or multiple and their size is usually between 0.5 and 5 mm, although the giant stone with several centimeters have been described like on our patient. The prostatic calculi may be associated with many diseases among them include benign prostate hyperplasia, adenocarcinoma, genitourinary tuberculosis and chronic infection [4, 5]. The calculations essentially sit on the periphery of the gland, in contact with the prostatic capsule.

Patients can present with lower urinary tract symptoms, urinary retention, pain, urethral strictures, or symptomatic urethral stones. Chronic prostatitis and recurrent urinary tract infections have been implicated in their development [6]. Such infection and stasis likely results in increased pressure and often results in “autoprostatectomy” of the prostate tissue [7]. Enucleation of the gland is therefore seldom necessary as part of stone management. The prostatic calculi can been often asymptomatic and discovered incidentally during the assessment of pathologies frequently associated, such as benign prostatic hyperplasia (BPH) and prostatitis, which constitute the major contributing factors [8, 9].

Clinical examination is usually normal but sometimes these calculations are collected on DRE as a hard nodule that was suspected more of a prostate cancer. The diagnosis of prostatic calculi is radiological standard radiographs or ultrasound especially prostate transrectal which has better diagnostic sensitivity [10].

The asymptomatic prostatic calculi, the most common case, do not require treatment. When symptomatic, treatment is that of the associated urological condition. In case of chronic recurrent prostatitis, antibiotic treatment is often inadequate and endoscopic resection of the prostate is sometimes necessary. For large stones that can be extracted during a transurethral resection of the prostate, it is possible to associate the resection of a flush load in the bladder, where it can then be fragmented by lithotripsy in situ. It is also possible for very large calculations, to achieve a first in open surgery via open surgery, [11] such as a cystolitothomy in current case or an open “prostatolithotomy”

Other management techniques for giant prostatic calculi have included radical prostatectomy, cystotomy with bladder neck incision, and endoscopic lithotripsy [12]. Vesical neck incision has been recommended as an adjunct to relieve obstruction and improve drainage of local infection [4].

## Conclusion

Giant prostatic calculi are uncommon, usually associated with obstruction voiding, their diagnosis is easy. Infrared analysis revealed that majority of the prostatic calculi were mainly composed of calcium phosphates. Several management techniques for giant calculi were described.
